# Spatial variation of life-history traits in *Bulinus truncatus*, the intermediate host of schistosomes, in the context of field application of niclosamide in Côte d’Ivoire

**DOI:** 10.1186/s40850-021-00104-7

**Published:** 2022-01-21

**Authors:** Cyrille K. Konan, Yves-Nathan T. Tian-Bi, Nana R. Diakité, Mamadou Ouattara, Jean T. Coulibaly, Diabaté Salia, Amani Koné, Adolphe K. Kakou, Rufin K. Assaré, Mocket A. Ehouman, Sonya C. Glitho, Eliézer K. N’Goran

**Affiliations:** 1 Unité de Formation et de Recherche Biosciences, Université Félix Houphouët-Boigny, 22 BP 770 Abidjan 22, Côte d’Ivoire; 2 Centre Suisse de Recherches Scientifiques en Côte d’Ivoire, 01 BP 1303 Abidjan 01, Côte d’Ivoire; 3 Swiss Tropical and Public Health Institute, P.O. Box, CH-4002, Basel, Switzerland; 4 University of Basel, P.O. Box, CH-4003, Basel, Switzerland; 5 Centre d’Entomologie Médicale et Vétérinaire, Université Alassane Ouattara de Bouaké, 27 BP 529 Abidjan 27, Côte d’Ivoire; 6 Institut National d’Hygiène Publique, Ministère de la Santé et de l’Hygiène Publique, Boulevard Du Port (Chu)-Treichville, Bp V 14, Abidjan, Côte d’Ivoire

**Keywords:** Spatial variation, Life-history traits, *Bulinus truncatus*, Niclosamide treatments, Côte d’Ivoire

## Abstract

**Background:**

Control of intermediate host snails using molluscicides for the control and/or elimination of schistosomiasis is a strategy in line with WHO recommendations. Niclosamide is the main chemical molluscicide recommended by WHO. However, except the immediate killing of the snail, the extent of the impact of the molluscicide application on the evolution of snail life-history traits, in relation to recolonization of treated sites is not well known. This study aimed to characterize the spatial variation of life-history traits of populations of the freshwater snail *Bulinus truncatus*, in relation to niclosamide spraying in the field.

From 2016 to 2018, we conducted a trial, using niclosamide to control the intermediate host snails for interrupting the seasonal transmission of urinary schistosomiasis in northern and central Côte d’Ivoire. Five villages (sites) were considered, including three test and two control villages. In the test villages, the molluscicide was sprayed in habitats harboring *B. truncatus* snails three times a year (November, February–March and June). We sampled six *B. truncatus* populations: two populations from the control villages without any treatment; one collected before treatment and three sampled 2–3 months after treatment of the site with niclosamide. The snail populations were monitored for several life-history traits, including survival, growth, fecundity and hatchability, under laboratory conditions, over one generation (G_1_). We tested the population, region (North/Centre) and treatment status (treated/untreated) effects on the variation of the measured life-history traits and correlations between pairs of traits were estimated.

**Results:**

On the whole, the traits varied among populations. The risk of death was lower in northern populations compared to central ones. The age at first reproduction was reached earlier with a smaller size of snails in northern populations. Values of first reproduction features (size and fecundity) were lower in treated snail populations. The overall growth of untreated populations was higher than that of treated ones. The late fecundity and egg hatching were higher in northern than in central snails. At first reproduction, age was negatively correlated with some fecundity parameters. However, growth was positively associated with fecundity.

**Conclusions:**

Our study showed a spatial variation of life-history traits in *B. truncatus* snails. The mollusciciding seems to have led to the depression of some life-history traits in the snail populations. However, investigations should be carried out over several generations of snails to better clarify the impact of niclosamide on the evolution of the life-history traits.

**Supplementary Information:**

The online version contains supplementary material available at 10.1186/s40850-021-00104-7.

## Background

Schistosomiasis is the most important snail-borne disease in tropical countries, with more than 90% of cases occurring in sub-Saharan Africa [1–3]. Despite increasing efforts to control this parasitic disease, it still remains a public health concern in endemic countries, affecting approximately 260 million people [3] and leading to serious economic losses [4].

Transmission occurs in freshwater bodies, used intensively by both humans and livestock, where intermediate host snails and their associated schistosomes are present [5, 6]. Hence, the distribution of genera and species of intermediate host snails compatible with the schistosome parasite influences the distribution of the disease. Schistosome transmission cannot occur without compatible snails [7].

The World Health Assembly (WHA) recommended local schistosomiasis elimination “where feasible” to member countries, through the resolution WHA65.21 [8]. Control of intermediate host snails using molluscicides for the control and/or elimination of schistosomiasis is a strategy in line with WHO recommendations [9]. Niclosamide (Bayluscide®) is the main molluscicide registered and recommended as effective by WHO [10]. For example, schistosomiasis control projects conducted in Morocco, China and Egypt showed that snail control using this molluscicide could be an efficient approach for reducing or interrupting the transmission of schistosomes [11–13]. Moreover, recent systematic reviews and meta-analyses on the impact of the application of chemical molluscicides [14, 15] demonstrate the importance of integrating snail control using niclosamide in schistosomiasis elimination campaigns in endemic areas [7].

Like all living organisms, host snail populations are known to vary for biological traits under the combination of genetic and environmental factors [16–18]. Effective control of these snails, therefore, requires a deep knowledge of their life cycle [19]. For this purpose, it is necessary to assess the variation of life-history traits of populations [20, 21]. These traits include growth, survival and reproduction, with age at first reproduction being one of these organizer parameters [22].

In this context, freshwater gastropods of the Hygrophila group offer excellent biological model. The snail *Bulinus truncatus* is one of these gastropods. In Africa and the Middle East, this snail acts as intermediate host for both *Schistosoma haematobium* and *S. bovis*, the agents of human and cattle schistosomiasis, respectively [23, 24]. In Côte d’Ivoire, *B. truncatus* is the main intermediate host for those parasites in northern and central regions. The snail is a hermaphroditic selfer, with selfing rates up to 80% in some populations [25]. Life-history traits can vary within and among populations of *B. truncatus* [26–28].

Previous studies reported that niclosamide is highly active at all stages of the life cycle of freshwater snails, killing them within a few hours at low concentrations [7, 14, 15]. However, except the immediate killing of the snail, the extent of the impact of the molluscicide application on the evolution of snail life-history traits, in relation to recolonization of treated sites is not well known. Nonetheless, pesticides are known to impact several biological traits of organisms [29, 30]. For instance, chlorpyrifos® and profenophos® were shown to reduce survival rate, growth rate and egg production, and to cause severe damages in the hermaphroditic gland cells of *B. truncatus* snails [31]. Therefore, niclosamide may influence the evolution of life traits. It is thus important to know how life-history traits vary in *B. truncatus* populations in relation to habitat treatment with the molluscicide. We monitored several traits in six natural populations of the snail, over one generation, under laboratory conditions, using a family design. We characterized the spatial variation of life-history traits in these populations, in relation to niclosamide application in the field.

This study is part of a Schistosomiasis Consortium for Operational Research and Evaluation (SCORE) large-scale project [32] that aimed to interrupt seasonal transmission of *S. haematobium* in northern and central regions of Côte d’Ivoire by combining chemical snail control using niclosamide and mass drug administration (MDA) with praziquantel [33].

## Results

### Survival of populations

**Fig. 1:**
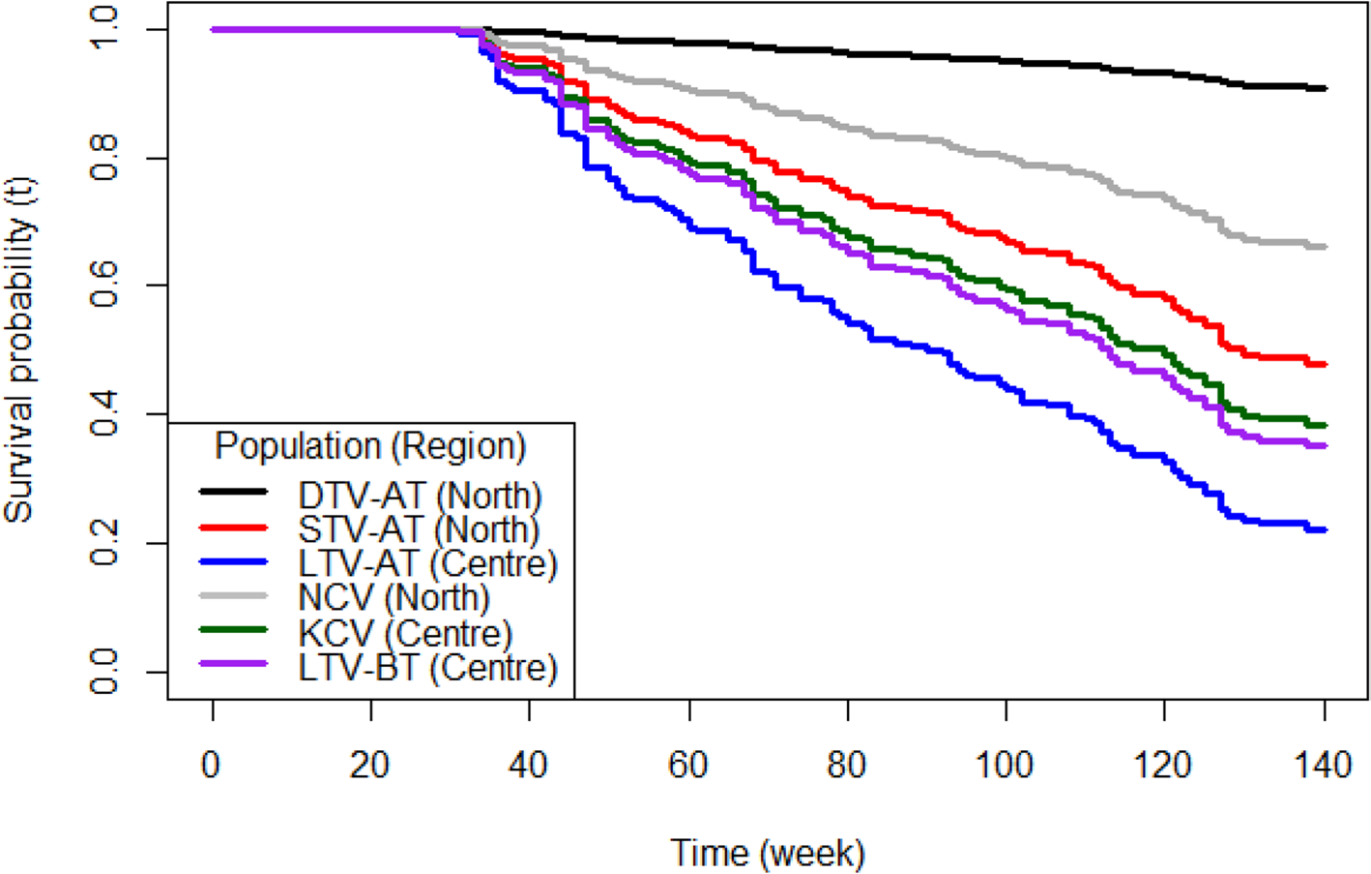
Survival curves of *Bulinus truncatus* populations collected in five different locations distributed in two regions according to treatment. DTV-AT, STV-AT and LTV-AT are Djemitedouo, Sambakaha and Linguebo populations collected 2–3 months after niclosamide application, respectively. NCV and KCV are Noumousso and Kongobo populations from locations never treated, respectively. LTV-BT is Linguebo population collected before treatment. Abbreviations: DTV-AT = Djemitedouo test village-after treatment, STV-AT = Sambakaha test village-after treatment, LTV-AT = Linguebo test village-after treatment, LTV-BT = Linguebo test village-before treatment, NCV = Noumousso control village and KCV = Kongobo control village

Populations varied for survival rate according to the region and the treatment status (Fig. 1). At the end of the experiment, the survivorship of populations collected 2–3 months after niclosamide application, from Djemitedouo (DTV-AT), Sambakaha (STV-AT) and Linguebo (LTV-AT), were 91.11, 48.89 and 17.78%, respectively. In the population of Linguebo before treatment (LTV-BT) and never treated populations of Noumousso (NCV) and Kongobo (KCV), the survival rates were 42.22, 64.44 and 35.56%, respectively. Region effect on the risk of death was highly significant (*p* <  0.001), with a factor of 3.25 per week. Hence, this factor was 0.30 time lower in the northern region than in the central one. Treatment effect on the risk of death was not significant (*p* > 0.05). However, the risk of death was increased by a factor of 1.28 (with β > 0), resulting in 0.78 time lower in the untreated than in the treated group (Table 1).

**Table 1:** Modeling to the survival of *Bulinus truncatus* populations according to the region and the treatment status using Cox proportional hazards regression

	β	Exp (β)	***p***-value	95% CI
Central region	1.179	3.251	< 0.001	2.211, 4.781
Treated group	0.248	1.281	0.175	0.896, 1.832

β is the Cox regression coefficient. Exp (β) represents the hazard ratio (HR) or risk of death. HR = 0: No effect, HR < 1: Reduction in the hazard, HR > 1: Increase in the hazard

### First reproduction parameters of populations

Significant differences were detected between regions for age and size at first reproduction, only. Compared to central populations, the age at first reproduction was reached earlier with a smaller size of snails in northern populations (GLM, *p* <  0.001; Table 2). No significant variation was observed for fecundity at first reproduction between regions. However, size and, numbers of egg capsules, eggs and hatched eggs varied according to the treatment status. The values of these parameters were lower in the treated group than in the untreated one (Table 2). In addition, numbers of eggs, eggs per capsule and hatched eggs were lower in Linguebo test village-after treatment (LTV-AT) than in Linguebo test village-before treatment (LTV-BT) (Additional file: Table S3).

**Table 2:** Variation of first reproduction parameters in *Bulinus truncatus* populations according to the region and the treatment status

Traits	Region		Treatment		***p***_**reg**_	***p***_**trt**_
	North	Centre	Treated	Untreated		
Age.1rep ± SD	69.23 ± 11.51^**a**^	87.49 ± 18.22^**b**^	74.92 ± 17.92^**a**^	76.45 ± 15.33^**a**^	< 0.001	0.249
Size.1rep ± SD	3.51 ± 0.42^**a**^	3.84 ± 0.31^**b**^	3.50 ± 0.47^**a**^	3.75 ± 0.30^**b**^	< 0.001	< 0.001
NEC.1rep ± SD	2.37 ± 1.30^**a**^	2.31 ± 1.41^**a**^	2.11 ± 1.27^**a**^	2.59 ± 1.36^**b**^	0.823	0.042
NE.1rep ± SD	5.56 ± 3.69^**a**^	5.72 ± 4.30^**a**^	4.69 ± 3.68^**a**^	6.58 ± 3.92^**b**^	0.674	< 0.001
NEPC.1rep ± SD	2.32 ± 0.85^**a**^	2.46 ± 0.99^**a**^	2.18 ± 0.91^**a**^	2.56 ± 0.86^**a**^	0.576	0.105
Hatch.1rep ± SD	2.04 ± 0.49^**a**^	2.64 ± 0.15^**a**^	1.61 ± 0.36^**a**^	2.92 ± 0.97^**b**^	0.338	< 0.001

Age.1rep, Size.1rep, NEC.1rep, NE.1rep, NEPC.1rep and Hatch.1rep are age, size, mean number of egg capsules, eggs and eggs per capsule and hatching rate at first reproduction. *P*_reg_ refers to the *p*-value associated with region effect. *P*_trtm_ refers to *p*-value associated with treatment effect. Mean ± SD of the same row with the same superscript letter are not significantly different

Correlation tests between first reproduction parameters showed both positive and negative coefficients, on the whole (Additional file 1: Table S1). Age was negatively correlated with the number of egg capsules and eggs, whatever the group considered. A positive association was found between size and number of eggs per capsule except in the central region. Similar positive correlations were detected between size and numbers of eggs laid in the northern region (Table S1).

### Growth, fecundity and egg hatching of populations

Overall, the best-fitting model to describe the life-history trait evolution of the snail populations, according to the region and treatment effects, was that with both random intercept and slope.

**Fig. 2:**
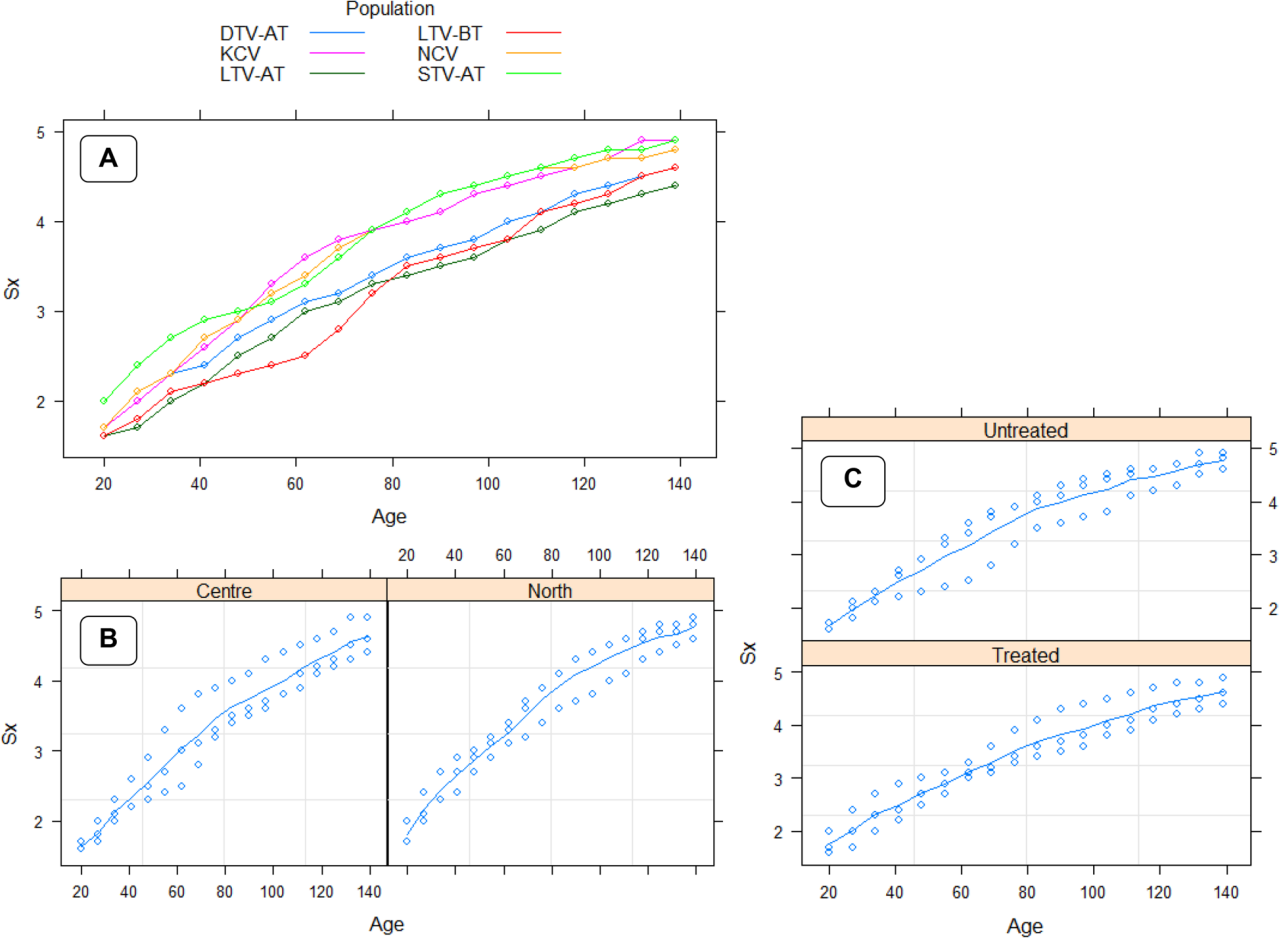
Growth evolution of *Bulinus truncatus* populations. **a**. In the six studied populations. **b**. In the two regions. **c**. For the two treatment statutes. Sx in mm is mean size / snail / week. Age is in days. DTV-AT, STV-AT and LTV-AT are Djemitedouo, Sambakaha and Linguebo populations collected 2–3 months after niclosamide application, respectively. NCV and KCV are Noumousso and Kongobo populations from locations never treated, respectively. LTV-BT is Linguebo population collected before treatment. Abbreviations: DTV-AT = Djemitedouo test village-after treatment, STV-AT = Sambakaha test village-after treatment, LTV-AT = Linguebo test village-after treatment, LTV-BT = Linguebo test village-before treatment, NCV = Noumousso control village and KCV = Kongobo control village

There was a variation of growth evolution among populations (Fig. 2a). At regional scale, growth intercept (initial size) was significantly higher in northern populations than in central ones (t-test; t = 5.23, *p* < 0.01). On the other hand, no difference was observed between the North and the Centre, for growth slope (*p* > 0.05; Fig. 2b, Table 3). The effect of treatment was significant on growth slope (late size) only (t = 4.50, *p* < 0.05; Fig. 2c, Table 3), with higher value observed in untreated populations.

**Table 3:** Variation of intercept and slope of growth evolution in *Bulinus truncatus* populations according to the region and the treatment status

	Intercept ± SD	Slope ± SD
**Region**		
North	1.663 ± 0.093^**b**^	0.024 ± 0.001^**a**^
Centre	1.339 ± 0.093^**a**^	0.025 ± 0.001^**a**^
**Treatment**		
Treated	1.547 ± 0.102^**a**^	0.023 ± 0.001^**a**^
Untreated	1.454 ± 0.102^**a**^	0.026 ± 0.001^**b**^

Values in the same column with the same superscript letter are not significantly different

**Fig. 3:**
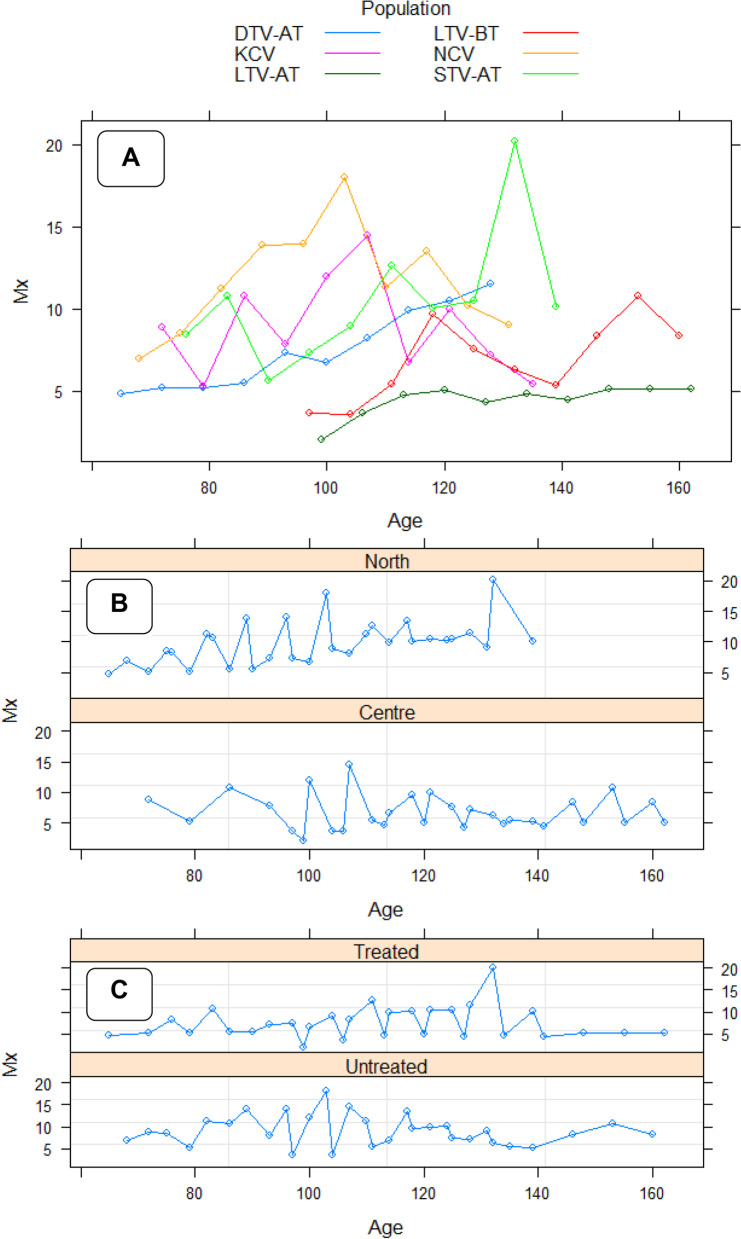
Fecundity evolution of *Bulinus truncatus* populations. **a**. In the six studied populations. **b**. In the two regions. **c**. For the two treatment statutes. Mx is mean number eggs / snail / week. Age is in days. DTV-AT, STV-AT and LTV-AT are Djemitedouo, Sambakaha and Linguebo populations collected 2–3 months after niclosamide application, respectively. NCV and KCV are Noumousso and Kongobo populations from locations never treated, respectively. LTV-BT is Linguebo population collected before treatment. Abbreviations: DTV-AT = Djemitedouo test village-after treatment, STV-AT = Sambakaha test village-after treatment, LTV-AT = Linguebo test village-after treatment, LTV-BT = Linguebo test village-before treatment, NCV = Noumousso control village and KCV = Kongobo control village

The fecundity of *B. truncatus* populations showed a strong variability between regions (Fig. 3a). The intercept of fecundity evolution was higher in the Centre than in the North (t = 3.75, *p* < 0.05). In contrast, the slope obtained in the Centre was lower with a negative value (t = 6.78, *p* < 0.01; Fig. 3b; Table 4). No difference was detected between treated and untreated groups, for both the intercept and the slope (t-test, *p* > 0.05; Fig. 3c, Table 4).

**Table 4:** Variation of intercept and slope of fecundity evolution in *Bulinus truncatus* populations according to the region and the treatment status

	Intercept ± SD	Slope ± SD
**Region**		
North	1.035 ± 2.784^**a**^	0.087 ± 0.026^**b**^
Centre	8.152 ± 2.907^**b**^	−0.011 ± 0.023^**a**^
**Treatment**		
Treated	5.608 ± 3.032^**a**^	0.016 ± 0.026^**a**^
Untreated	10.978 ± 3.095^**a**^	−0.016 ± 0.027^**a**^

Values in the same column with the same superscript letter are not significantly different

**Fig. 4:**
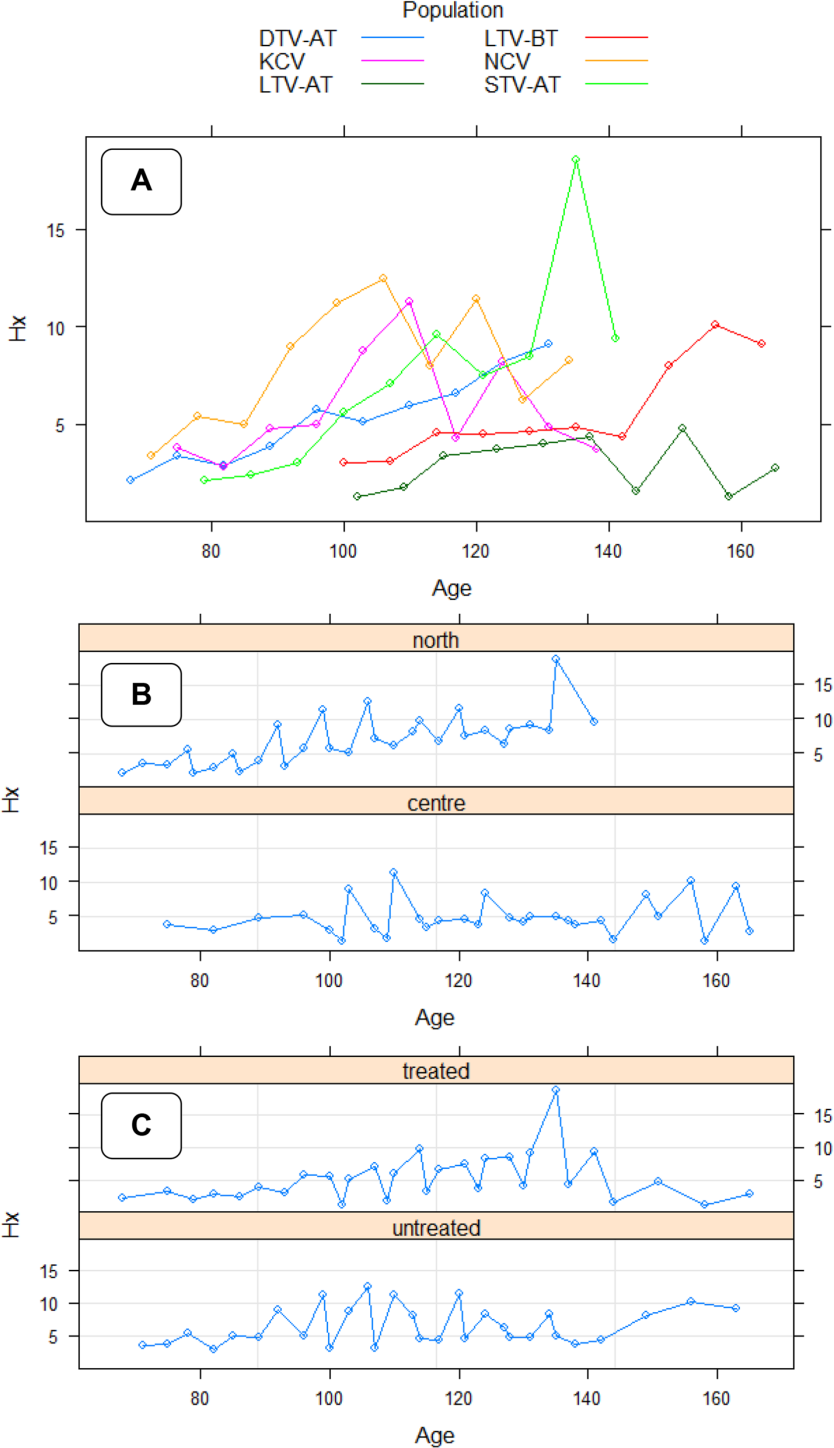
Egg hatching evolution of *Bulinus truncatus* populations. **a.** In the six studied populations. **b.** In the two regions. **c.** For the two treatment statutes. Hx is mean number hatched eggs / snail / week. Age is in days. DTV-AT, STV-AT and LTV-AT are Djemitedouo, Sambakaha and Linguebo populations collected 2–3 months after niclosamide application, respectively. NCV and KCV are Noumousso and Kongobo populations from locations never treated, respectively. LTV-BT is Linguebo population collected before treatment. Abbreviations: DTV-AT = Djemitedouo test village-after treatment, STV-AT = Sambakaha test village-after treatment, LTV-AT = Linguebo test village-after treatment, LTV-BT = Linguebo test village-before treatment, NCV = Noumousso control village and KCV = Kongobo control village

Similarly, egg hatching evolution greatly varied among populations (Fig. 4a). The intercept of hatching was significantly higher in central populations (t = 4.96, *p* < 0.01), whereas the slope was higher in northern ones (t = 7.17, *p* < 0.01; Fig.4b, Table 5). Both the intercept and the slope of hatching evolution did not vary according to the treatment status (t-test, *p* > 0.05; Fig. 4c, Table 5).

**Table 5:** Variation of intercept and slope of hatching evolution in *Bulinus truncatus* populations according to the region and the treatment status

	Intercept ± SD	Slope ± SD
**Region**		
North	−5.659 ± 2.459^**a**^	0.120 ± 0.023^**b**^
Centre	2.624 ± 2.548^**b**^	0.017 ± 0.020^**a**^
**Treatment**		
Treated	0.999 ± 2.786^**a**^	0.036 ± 0.023^**a**^
Untreated	2.871 ± 2.843^**a**^	0.031 ± 0.024^**a**^

Values in the same column with the same superscript letter are not significantly different

Significant correlations between traits at early and at late stages were all positive, whatever the group considered (Additional file 1: Table S2).

## Discussion

This is one of the first studies assessing niclosamide impact following field treatments on the evolution of life-history traits in populations of the schistosome host snail, *B. truncatus*, under laboratory conditions. We discuss below what the analysis of variation of populations’ survival and, of the features of other traits at different life stages and overall life cycle bring to our understanding of these population functioning, in relation to chemical mollusciciding.

### Survivorship

Our findings showed no effect of treatment but a region effect on the variation of population survivorship, with a higher survival rate in the northern region. This result might indicate a better adaptation of northern snail populations to their environmental conditions compared to those from the Centre. Indeed, the climatic conditions appear relatively harsher and more variable in the North of the country. For example, the temperature ranges between 25 and 29 °C in the North versus 24–27 °C in the Centre. Likewise, there is a greater range of monthly rainfall in the North (3–300 mm of rain) compared to the central part of the country (11–200 mm) [34]. Consistently, previous studies have shown that populations living in unstable (variable) environments have a better adaptive capacity (supported by genetic diversity) than populations from stable environments [35–37].

### First reproduction features and life-history traits dynamics

In this study, the age at first reproduction was reached earlier with a smaller size of snails in northern populations compared to central ones. Previous works have already demonstrated differences in age and size at first reproduction among populations of vertebrates (e.g., mosquitofish *Gambusia affinis*: [38] and bird *Pyrrhocorax pyrrhocorax*: [39]) as well as of invertebrates such as freshwater snails, whether outcrossers [40] or selfers [41]; and these traits have been shown as key organizer parameters of the animal life cycle [42, 43]. A plausible explanation for the differences in these traits between regions could be that there is a greater fluctuation in water availability in the North than in the Centre. This may have favoured reproductively precocious snail individuals. Interestingly, size and, numbers of egg capsules, eggs and hatched eggs, at first reproduction, were lower in the treated group than in the untreated one. With regard to the SCORE project aiming at interrupting seasonal transmission of *S. haematobium*, this result may be considered as a good outcome of the mollusciciding. In fact, this suggests that, in the test villages, the snails swept away by treatments were replaced by smaller and less fertile individuals. This assumption was confirmed, for fecundity parameters, by the fact that in the Linguebo test village, the population collected after treatment (LTV-AT) displayed lower values than that sampled before treatment (LTV-BT). Moreover, larger host snails are known to produce more eggs per capsule and more cercariae (when infected) [44–46]; so the force of schistosome transmission would be lower with smaller snails.

Negative associations between age and some fecundity parameters (namely numbers of egg capsules and eggs) were detected whatever the factors considered (region or treatment). This indicates that the younger the snail, the higher its fecundity at first reproduction. Similar results have been found in snails such as *Biomphalaria pfeifferi*, another selfer [47] and *Physa acuta*, an outcrosser [48]. This result could be explained by the fact that the longer individuals take to make their first reproduction, the more resources would be allocated to other functions such as survival, although no correlation between this trait and fecundity at first reproduction was found in this study (result not shown). The positive correlation found between size and fecundity (number of eggs and of eggs per capsule) is consistent with previous studies on freshwater snails [41, 45], indicating that larger individuals lay more eggs.

In this study, all correlations between traits (size and, numbers of egg capsules, eggs, eggs per capsule and hatched eggs) at early and at late stages were positive, whatever the group considered. This is consistent with the correlation observed between size and fecundity parameters at first reproduction. This finding might suggest that between these two groups of traits, the distribution of resources is proportionally maintained during the life cycle of populations, as indicated by van Noordwijk and de Jong [49]. Moreover, Dillon reported that reproductive effort is generally a function of adult size in most freshwater snails [50].

The analysis of life trait dynamics showed that growth intercept (initial size) was higher for northern populations than for central ones; however, no difference was detected for growth slope (late size). This might indicate that during the monitoring of snails in the laboratory, individuals from the Centre used the available resources more for growth than for other functions (e.g., survival, fecundity), compared to individuals from the North. This may be explained by the lower survival rate and fecundity slope in the central populations, reflecting a trade-off between growth, survival and fecundity in these populations [51–53]. On the other hand, growth slope was lower in treated populations compared to that in untreated ones. Once again, this might be a result of mollusciciding. However, the actual effect of chemical treatments in such trait depressions observed should be further investigated.

## Conclusions

This study revealed variations in life-history traits (namely survival, growth and fecundity) between the *Bulinus truncatus* populations studied and according to the origin (North/Centre) of these populations. Furthermore, the mollusciciding seems to have caused some depression of life-history traits in the snail populations from test sites. However, further investigations conducted over several snail generations would better clarify the impact of niclosamide application on the evolution of *B. truncatus* life-history traits.

## Methods

### Study area

**Fig. 5:**
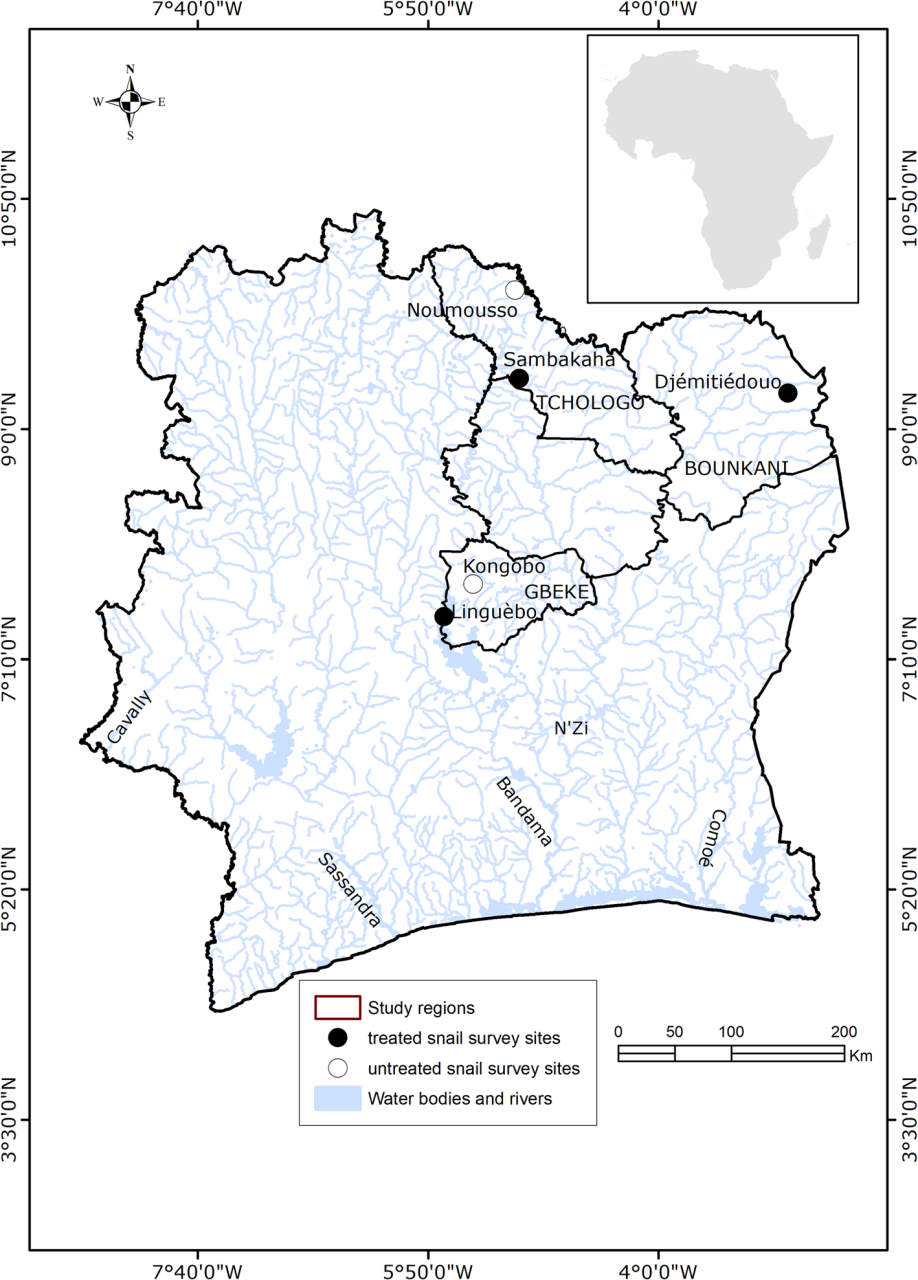
Map of Côte d’Ivoire showing the location of the five villages included in the study. The names of the village are indicated in normal character and names of the regions are in capital. Data are merged for all surveys conducted between March 2017–June 2018

This study was carried out in the North (Bounkani and Tchologo regions) and in the Centre (Gbêkê region) of Côte d’Ivoire (Fig. 5). These areas are known to harbour *B. truncatus* [54–56] which is predominantly involved in the transmission of *S. haematobium*, *S. bovis* and the *S. haematobium* x *S. bovis* hybrid [57]. In those regions, the snail is mainly observed in lakes of man-made dams, which are subject to a marked seasonality in relation to the climate [54]. These regions have virtually the same climate qualified as a savannah [58]. In northern Côte d’Ivoire, the temperature ranges from 25 °C to 29 °C (mean: 26.8 °C) and the monthly precipitation varies between 3 mm and 300 mm (annual mean: 1300 mm). In the central part, the values are 24 °C to 27 °C (mean: 26.2 °C) and 11 mm to 200 mm (annual mean: 1100 mm), respectively [34]. The dry season lasts from November to March, though a quite high rainfall is recorded in March in the central part of the country. In the northern area, two test villages (sites), namely Djémitédouo (DTV) (09°17′05.87″N, 02°58′08.81″W) and Sambakaha (STV) (09°24′10.72″N, 05°06′21.24″W) and one control village (site), Noumousso (NCV) (10°06′25.67″N latitude, 05°08′30.81″W longitude), were considered. In the central part, one test village (site), Linguèbo (LTV) (07°30′16.36″N, 05°42′22.60″W) and one control village (site), Kongobo (KCV) (07°45′50.33″N, 05°28′33.99″W), were selected. Of note, the test villages were those where both chemical snail control with niclosamide and MDA with praziquantel were applied and the two control villages were randomly chosen among the villages of other intervention arms with MDA only, from the whole SCORE project.

### Snail sampling

**Fig. 6:**
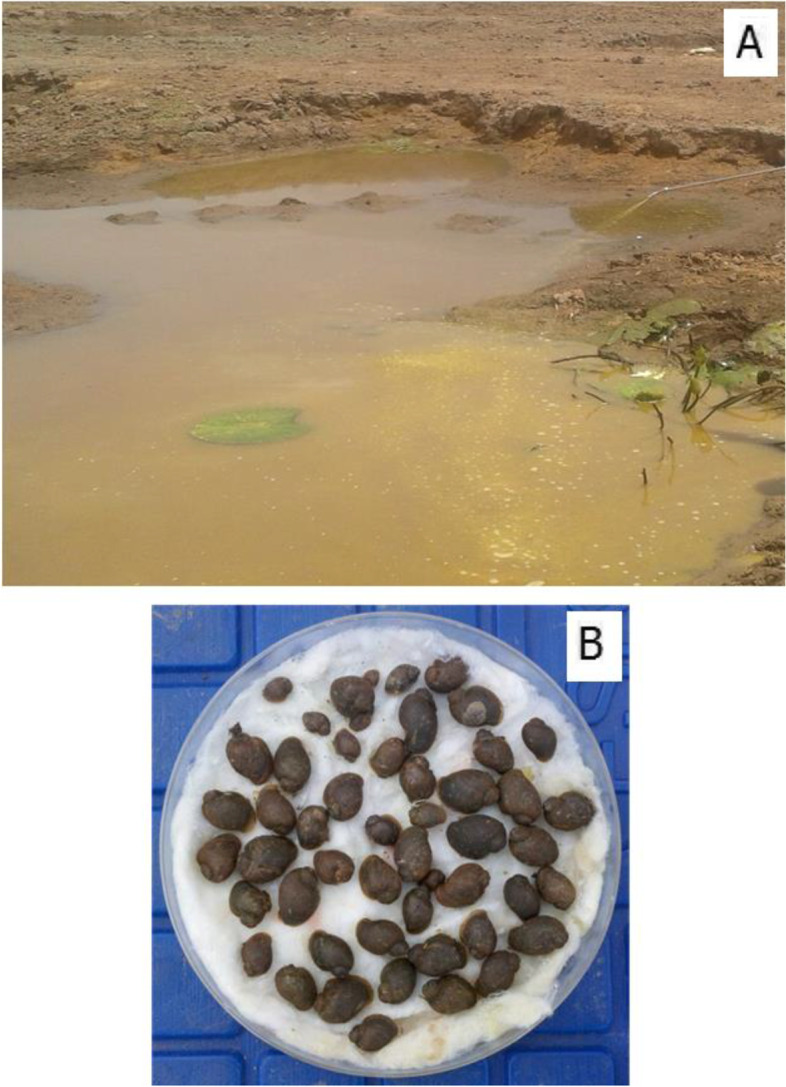
**a** Niclosamide sprayed against *Bulinus truncatus* in a site of Korokara, northern of Côte d’Ivoire. **b** Snails alive placed in a petri dish according to Sellin and Roux (1974)

In each study village, human-water contact sites were identified and georeferenced using a hand-held global positioning system (GPS; Garmin Sery GPS MAP 62, Olathe, KS, USA) device [33]. Snail surveys were carried out at each water contact point in March 2017, June 2017, November 2017, March 2018 and June 2018. Before each snail sampling, physicochemical parameters of the water were measured in situ using a pocket multi-parameter tester (HANNA® Instruments HI 98129 Combo, Woonsocket, RI, USA). The parameters measured were temperature, pH, conductivity and total dissolved solids (TDS). Snails were collected by the same two experienced collectors, using a long-handled scoop and/or forceps for a period of 15 min [41, 59]. Shortly after each sampling, snails were morphologically identified on the basis of standard identification keys to the species level [23, 60] and counted. In test villages (sites), when *B. truncatus* snails were found, an appropriate volume of niclosamide solution (at concentration of 10 g/l) was applied along the bank of the water body (Fig. 6a).

The populations studied were composed of snail individuals collected, before treatment with niclosamide, from the site of the test village Linguèbo (LTV-BT), individuals from the sites recolonized 2–3 months after treatment, of the test villages Djemitedouo (DTV-AT), Sambakaha (STV-AT) and Linguebo (LTV-AT) and, snails from sites of the control villages Noumousso (NCV) and Kongobo (KCV). The *B. truncatus* snails collected (G_0_ generation) were placed between two layers of moistened cotton in a labeled petri dish and transferred to the laboratory [61] (Fig. 6b).

### Experimental design

**Fig. 7:**
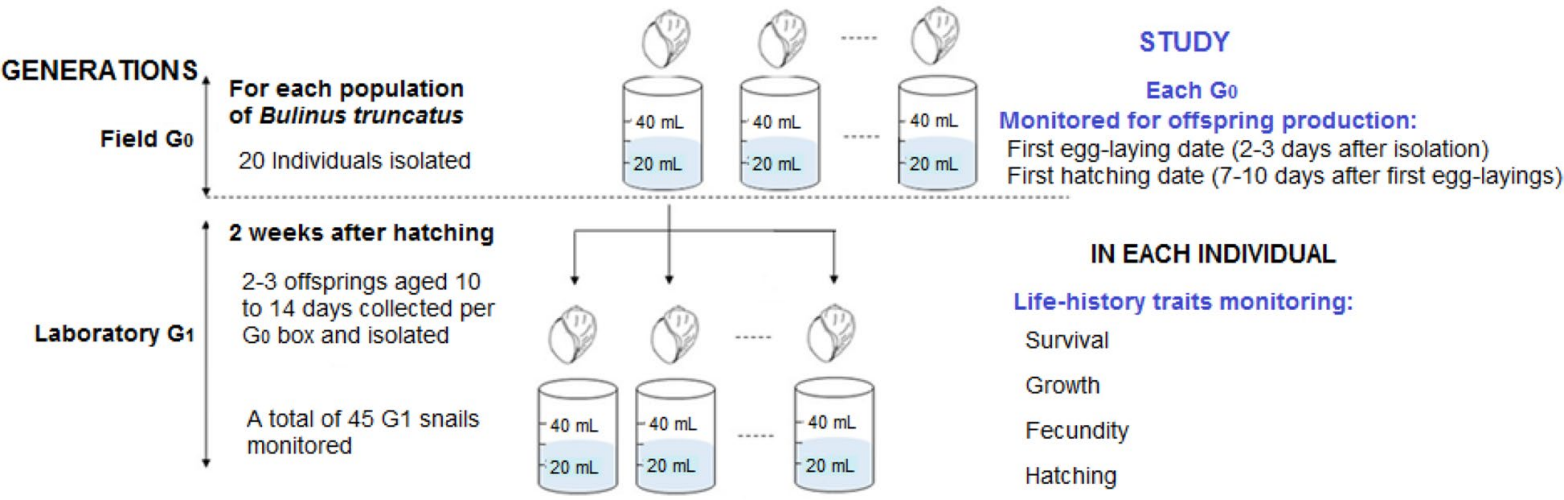
Schematic diagram of the experimental protocol for the study of the life-history traits

The experimental protocol of this study is indicated in Fig. 7. Snails were reared following a *common garden* approach, where individuals from different sites were raised together in the same controlled (laboratory) conditions [62]. The rearing room and water (30 mL of mineral water) were maintained at a temperature of 22–24 °C and under a photoperiod of 12 L/12 D. Adult G_0_ snails were fed ad libitum with granules for aquarium fish and juveniles with boiled lettuce. Water was changed and food given twice a week, in rearing boxes.

In the laboratory, G_0_ snails from the same site were put together in a 1.5 L transparent plastic tanks for acclimatization. The following day, 20 G_0_ individuals of each population were randomly chosen. Each G_0_ snail was isolated in a 40 mL transparent plastic box filled with 30 mL of mineral water for G_1_ offspring production. Two or 3 days later, egg-layings were observed synchronously in the boxes. After hatching, from 7 to 10 days after the first egg-laying, the offspring were maintained with the parents in rearing boxes for approximately 2 weeks. Then, two or three G_1_ offspring, aged 10–14 days were chosen at random in each box, giving a total of 45 G_1_ snails for each population. These G_1_ individuals were isolated and reared in the same rearing conditions as their parents.

### Assessment of life-history traits

Life-history traits of G_1_ individuals were monitored over 28 weeks. These traits included survival, fecundity, growth and hatching rate. Size (shell height) was measured once a week using a graph paper under binocular magnifying glass. This allowed the estimation of size at first reproduction, and growth. Individual survival was monitored three times a week by observation with the naked eye. Fecundity of each individual snail was evaluated by checking egg-layings twice a week: number of egg capsules, eggs and eggs per capsule were the parameters considered. Age and fecundity at first reproduction were recorded. Fecundity at first reproduction was defined as the number of egg capsules, eggs and eggs per capsule at the first egg-laying. The numbers of egg capsules as well as eggs laid over 10 weeks were counted using a binocular magnifying glass and recorded. Fecundity at population level was also measured based on the parameter Mx (mean number of eggs / snail / week). Mx was computed according to Ragheb et al. and Habib et al. [63, 64]. Egg hatching was also monitored every 2 days over 2 weeks. At the end of each week, the egg capsules laid in each box were collected using a plastic spatula and put in a petri dish containing mineral water. Egg capsules were checked every 2 days over 2 weeks and the number of hatched individuals was recorded in order to compute the hatching rate per week for each population. Of note, snails were monitored for their life-history traits according to two life stages: the early stage (i.e., the period between the isolation of individuals and the second week after their first egg-laying) and the late stage (i.e., the period after the second week of egg-laying).

### Statistical analysis

First of all, the Kolmogorov-Smirnov test was used to assess the normal distribution of each measured variables. Region and treatment effects on survival of populations were tested using Cox proportional hazards regression. Variation of first reproduction parameters between regions and treatment statutes was tested using generalized linear models (GLM). These parameters were age, size and, numbers of egg capsules, eggs, eggs per capsule and hatched eggs. Pearson correlation test was performed to assess the relationship between traits. Repeated measure traits such as size, fecundity and hatchability were analyzed using a mixed linear model. For this purpose, three models were tested, namely model 1: random intercept, model 2: random slope and, model 3: random intercept and slope. The best-fitting model was chosen based on the lower value of the Bayesian Information Criterion (BIC). The t-test was used to evaluate the variation of intercepts and slopes between regions and treatment statutes. Repeated measures correlation was performed to test association between traits (size (growth) and, numbers of egg capsules, eggs, eggs per capsule and hatched eggs) measured at early and at late life stages according to the region and the treatment status.

All statistical analyses were performed using different packages of the software R version 3.6.1.

## Supplementary Information

**Additional file 1: Table S1**: Correlations between traits at first reproduction in G1 individuals from natural *Bulinus truncatus* populations according to the region and the treatment status. **Table S2**: Correlation coefficients for repeated measures between traits at early and late stages for the two study regions and the two treatment statutes. **Table S3**: Variation of first reproduction parameters of the *Bulinus truncatus* populations collected before and after niclosamide application in the Linguebo test village.

## Data Availability

The data used and / or analyzed during this study are available from the corresponding author on reasonable request.

## Abbreviations

WHO: World Health Organization
WHA: World Health Assembly
SCORE: Schistosomiasis Consortium for Operational Research and Evaluation
MDA: Mass Drug Administration
GPS: Global Positioning System
pH: Potential Hydrogen
TDS: Total Dissolved Solids
DTV-AT, STV-AT and LTV-AT: populations from the Djemitedouo, Sambakaha and Linguebo test villages, respectively, 2–3 months after treatment with niclosamide
NCV and KCV: populations from the Noumousso and Kongobo control villages
LTV-BT: population from the Linguebo test village before niclosamide application
*p*: *p*-value
SD: Standard deviation
*P*reg: *p*-value associated to region effect
*P*trt: *p*-value associated to treatment effect
