# Supplementary material for: Spatial variation of life-history traits in Bulinus truncatus, the intermediate host of schistosomes, in the context of field application of niclosamide in Côte d’Ivoire
Source: BMC Zool. 2022 Jan 21;7:7. doi: 10.1186/s40850-021-00104-7 (PMC10127057; doi:10.1186/s40850-021-00104-7)
Supplement: Supplementary file 1 — Additional file 1: Table S1: Correlations between traits at first reproduction in G1 individuals from natural Bulinus truncatus populations according to the region and the treatment status. Table S2: Correlation coefficients for repeated measures between traits at early and late stages for the two study regions and the two treatment statutes. Table S3: Variation of first reproduction parameters of the Bulinus truncatus populations collected before and after niclosamide application in the Linguebo test village. [file 40850_2021_104_MOESM1_ESM.docx]

**Table S1:** Correlations between traits at first reproduction in G_1_ individuals from natural *Bulinus truncatus* populations according to the region and the treatment status

| **Region** | **Age.1rep** | **Size.1rep** | **NEC.1rep** | **NE.1rep** | **NEPC.1rep** | **Hatch.1rep** |
| --- | --- | --- | --- | --- | --- | --- |
| **Age.1rep** | — | 0.19* | - 0.30* | - 0.20* | 0.05 | - 0.23* |
| **Size.1rep** | 0.02 | — | 0.08 | 0.29* | 0.48*** | 0.17 |
| **NEC.1rep** | - 0.40* | 0.06 | — | 0.80*** | 0.02 | 0.68*** |
| **NE.1rep** | - 0.36* | 0.13 | 0.80*** | — | 0.52*** | 0.62*** |
| **NEPC.1rep** | 0.02 | 0.11 | 0.03 | 0.53*** | — | 0.16 |
| **Hatch.1rep** | - 0.15 | 0.15 | 0.30* | 0.58*** | 0.54*** | — |
|  |  |  |  |  |  |  |
| **Treatment** | **Age.1rep** | **Size.1rep** | **NEC.1rep** | **NE.1rep** | **NEPC.1rep** | **Hatch.1rep** |
| **Age.1rep** | — | 0.44*** | - 0.23* | - 0.25* | - 0.10 | - 0.24* |
| **Size.1rep** | 0.01 | — | 0.00 | 0.19 | 0.36*** | - 0.01 |
| **NEC.1rep** | - 0.42*** | 0.02 | — | 0.80*** | 0.07 | 0.65*** |
| **NE.1rep** | - 0.24* | 0.14 | 0.80*** | — | 0.57*** | 0.64*** |
| **NEPC.1rep** | 0.28* | 0.24* | - 0.11 | 0.43*** | — | 0.21* |
| **Hatch.1rep** | - 0.01 | 0.31* | 0.37*** | 0.54*** | 0.38*** | — |

Correlations between traits estimated in northern populations and in treated populations are shown above the main diagonal, and those estimated in central snails and in untreated snails are shown below the main diagonal. Age.1rep, Size.1rep, NEC.1rep, NE.1rep, NEPC.1rep and Hatch.1rep are age, size, mean number of egg capsules, eggs and eggs per capsule and hatching rate at first reproduction. Values observed are Pearson’s correlation coefficients.

**P* < 0.05; ****P* < 0.001.

**Table S2:** Correlation coefficients for repeated measures between traits at early and late stages for the two study regions and the two treatment statutes.

| **North** | **Size** | **NEC** | **NE** | **NEPC** | **Hatching** |
| --- | --- | --- | --- | --- | --- |
| **Size** | — | 0.47*** | 0.51*** | 0.52*** | 0.37*** |
| **NEC** | 0.32*** | — | 0.88*** | 0.55*** | 0.55*** |
| **NE** | 0.34*** | 0.85*** | — | 0.70*** | 0.67*** |
| **NEPC** | 0.35*** | 0.18*** | 0.47*** | — | 0.44*** |
| **Hatching** | 0.34*** | 0.66*** | 0.85*** | 0.41*** | — |
|  |  |  |  |  |  |
| **Centre** | **Size** | **NEC** | **NE** | **NEPC** | **Hatching** |
| **Size** | — | 0.30*** | 0.37*** | 0.45*** | 0.37*** |
| **NEC** | 0.06 | — | 0.88*** | 0.49*** | 0.63*** |
| **NE** | - 0.00 | 0.87*** | — | 0.57*** | 0.80*** |
| **NEPC** | 0.00 | 0.32*** | 0.53*** | — | 0.49*** |
| **Hatching** | 0.20* | 0.74*** | 0.81*** | 0.42*** | — |
|  |  |  |  |  |  |
| **Treated** | **Size** | **NEC** | **NE** | **NEPC** | **Hatching** |
| **Size** | — | 0.32*** | 0.29*** | 0.38*** | 0.33*** |
| **NEC** | 0.52*** | — | 0.87*** | 0.47*** | 0.52*** |
| **NE** | 0.52*** | 0.82*** | — | 0.60*** | 0.55*** |
| **NEPC** | 0.38*** | 0.28*** | 0.56*** | — | 0.35*** |
| **Hatching** | 0.45*** | 0.69*** | 0.90*** | 0.47*** | — |
|  |  |  |  |  |  |
| **Untreated** | **Size** | **NEC** | **NE** | **NEPC** | **Hatching** |
| **Size** | — | 0.52*** | 0.63*** | 0.63*** | 0.43*** |
| **NEC** | - 0.05 | — | 0.89*** | 0.57*** | 0.61*** |
| **NE** | - 0.05 | 0.89*** | — | 0.70*** | 0.78*** |
| **NEPC** | 0.07 | 0.19*** | 0.44*** | — | 0.51*** |
| **Hatching** | 0.13* | 0.68*** | 0.78*** | 0.35*** | — |

Correlations between traits at the early stage are shown above the main diagonal and those at the late stage are shown below the main diagonal. The early life stage refers to the period ranging from the date of isolation of the juvenile G_1_ snail’s isolation of the individuals and to the second week after their first egg-laying. The late life stage is the period from the second week after egg-laying to the death of snails. NEC = number of egg capsules; NE = number of eggs and NEPC = number of eggs per capsule.

**P* < 0.05; ****P* < 0.001.

**Table S3:** Variation of first reproduction parameters of the *Bulinus truncatus* populations collected before and after niclosamide application in the Linguebo test village

| **Traits** | **LTV-BT** | **LTV-AT** | **t** | ***p*-value** |
| --- | --- | --- | --- | --- |
| Age.1rep±SD | 97.60±13.09**^a^** | 100.73±13.40**^a^** | 0.691 | 0.495 |
| Size.1rep±SD | 3.84±0.33**^a^** | 3.89±0.34**^a^** | 0.407 | 0.687 |
| NEC.1rep±SD | 1.65±0.74**^a^** | 2.07±1.39**^a^** | 1.055 | 0.304 |
| NE.1rep±SD | 4.70±2.41**^b^** | 3.33±2.22**^a^** | - 1.963 | 0.049 |
| NEPC.1rep±SD | 2.94±0.94**^b^** | 1.66±0.65**^a^** | - 4.792 | < 0.001 |
| Hatch.1rep±SD | 3.45±1.36**^b^** | 1.00±1.60**^a^** | - 4.774 | < 0.001 |

Values of the same row with the same superscript letter are not significantly different. Abbreviations: Age.1rep = Age at first reproduction, Size.1rep = Size at first reproduction, NEC.1rep = Number of egg capsules at first reproduction, NE.1rep = Number of eggs at first reproduction, NEPC.1rep = Number of eggs per capsule at first reproduction, Hatch.1rep = Number of hatched eggs at first reproduction, LTV-BT = Linguebo test village-before treatment, LTV-AT = Linguebo test village-after treatment.
